# Decreased neuronal excitability in hypertriglyceridemia hamsters with acute seizures

**DOI:** 10.3389/fneur.2024.1500737

**Published:** 2024-12-19

**Authors:** Qiuyue Shen, Nana Liu, Yuwu Jiang, Lili Liu, Xinlin Hou

**Affiliations:** ^1^Department of Pediatrics, Peking University First Hospital, Beijing, China; ^2^Beijing Key Laboratory of Molecular Diagnosis and Study on Pediatric Genetic Diseases, Beijing, China; ^3^Key Laboratory for Neuroscience, Ministry of Education, National Health and Family Planning Commission, Peking University, Beijing, China; ^4^Center of Epilepsy, Beijing Institute for Brain Disorders, Beijing, China

**Keywords:** seizure, hypertriglyceridemia, ZDHHC14, transient outward K+ currents, palmitoylation modification

## Abstract

**Introduction:**

Neonatal seizures are the most common clinical manifestation of neurological dysfunction in newborns, with an incidence ranging from 1 to 5‰. However, the therapeutic efficacy of current pharmacological treatments remains suboptimal. This study aims to utilize genetically modified hamsters with hypertriglyceridaemia (HTG) to investigate the effects of elevated triglycerides on neuronal excitability and to elucidate the underlying mechanisms. The ultimate goal is to identify novel therapeutic targets for the treatment of neonatal seizures.

**Methods:**

Acute seizure models were established both *in vivo* and *ex vivo* using wild-type and Apolipoprotein C2 knockout (*Apoc2*^−/−^) hamsters. The frequency of tonic–clonic seizures was recorded. Excitatory postsynaptic potentials (EPSPs) and evoked action potentials (eAPs) of pyramidal neurons in the frontal cortex were measured. Fatty acid metabolomic analysis was conducted on microdialysate from the frontal cortex tissue post-seizure, and mRNA expression changes were also assessed.

**Results:**

*Apoc2*^−/−^ hamsters exhibited a reduced frequency of tonic–clonic seizures and diminished EPSP and eAP in comparison to wild-type hamsters. Following seizure induction, free palmitic acid levels in the frontal cortex dialysate significantly decreased, while the expression of palmitoyl acyltransferase 14 (ZDHHC14) in the frontal cortex tissue was higher in *Apoc2*^−/−^ hamsters than in wild-type hamsters. Additionally, the amplitude of transient outward potassium currents (I_A_) in cortical neurons of *Apoc2*^−/−^ hamsters was observed to be elevated compared to wild-type hamsters.

**Conclusion:**

Hypertriglyceridemic *Apoc2*^−/−^ hamsters exhibited reduced seizure frequency and decreased cortical neuron excitability. The upregulation of ZDHHC14, leading to increased I_A_, may be a crucial mechanism underlying the observed seizure protection.

## Introduction

Seizures are the most common clinical manifestation of neonatal neurological dysfunction, with an incidence rate of 1‰ to 5‰ (1). During the neonatal period, significant neurogenesis and synapse formation occur to meet the demands of neurodevelopment. The brain is in a physiologically active state, actively receiving excitatory signals (2, 3). Additionally, the immature nature of the developing brain during this period leads to an excitatory/inhibitory imbalance (4), which can be observed in the following three aspects: (i) Calcium channels that mediate excitatory inward currents develop early, while stabilizing excitatory potassium currents appear later (2). (ii) Excitatory glutamatergic neurons are heavily expressed during development, with the NMDA receptor GluN2B subunit expressed in various brain regions, promoting intracellular calcium influx and prolonging postsynaptic currents. (iii) Activation of GABA receptors in the early stages of development can cause chloride ion efflux, resulting in neuronal depolarization and increased excitability (5). Therefore, due to the unique physiology and vulnerability of the immature brain to damage, the incidence of seizures is higher in this age group than in others. Currently, the first-line treatment drug is phenobarbital, with an effectiveness rate of 50 to 64% in treating neonatal seizures (6). Uncontrolled seizures may lead to various neurological sequelae, including epilepsy, cerebral palsy, developmental delay, and psychomotor disorders (1, 7). Some studies have found that anti-seizure medications themselves can increase cellular apoptosis and potentially affect normal brain development by altering neurogenesis, synaptogenesis, cell proliferation, and migration (8–10). Consequently, there is still an ongoing search for new therapeutic targets in the treatment of neonatal seizures to improve the prognosis of affected infants.

A ketogenic diet has been shown to be significantly effective in the clinical treatment of intractable epilepsy. The characteristics of a ketogenic diet include high fat, low carbohydrates, and adequate protein. One type of ketogenic diet, known as the classic ketogenic diet, consists mainly of long-chain triglycerides, with approximately 80 to 90% of calories coming from fat. In this regimen, cellular metabolism relies on the oxidation of fatty acids as the primary source of energy (11). The mechanisms behind the reduction of seizures through a ketogenic diet are not fully understood. Existing research suggests that these mechanisms may be related to improvements in brain energy metabolism (12), neurotransmitter release (13), ion channels (14), and oxidative stress (15, 16). It is worth noting that whether triglycerides are involved in the neuroprotective effects during seizures remains unclear. Some clinical studies in adults have suggested that triglycerides may exert protective effects against cognitive impairment (17–19). However, research focusing on their role in neuronal excitability and their relevance during the neonatal period remains limited. To investigate this, we used a genetically edited hamster model with extreme hypertriglyceridemia to study the impact of high triglycerides on neuronal excitability and its underlying mechanisms. This research aims to provide new insights for identifying therapeutic targets for neonatal seizures.

This study utilized apolipoprotein C2 (*Apoc2*) knockout golden Syrian hamster models with hypertriglyceridemia. Apoc2 is a crucial component of chylomicrons, very low-density lipoprotein, and high-density lipoprotein, and it serves as an essential activator of lipoprotein lipase, which is necessary for triglyceride metabolism (20, 21). Consequently, *Apoc2*^−/−^ hamsters exhibit extremely high levels of triglycerides and cholesterol. Hamsters possess many lipid metabolic features similar to those of humans, such as a lipoprotein profile dominated by low-density lipoprotein and a high level of endogenous cholesteryl ester transfer protein expression (22), making them an ideal animal model for studying pathophysiological processes related to lipid metabolism. Furthermore, Apoc2 is almost absent in the brain, and the knockout of this gene does not affect neuronal function. Therefore, *Apoc2* knockout hamsters can be employed to investigate the role and mechanisms by which triglycerides and their metabolites contribute to neonatal seizures.

This study aims to elucidate the mechanisms through which elevated triglycerides modulate neuronal excitability and confer neuroprotection during seizures. By identifying the underlying pathways, this research seeks to uncover novel therapeutic targets for the treatment of neonatal seizures, offering new strategies to improve outcomes for affected infants.

## Methods

### Experimental animals

The animals used in this experiment were golden Syrian hamsters provided by Professors Yuhui Wang and Xunde Xian from the Institute of Cardiovascular Sciences and Key Laboratory of Molecular Cardiovascular Sciences, Peking University (purchased and bred at Beijing Vital River Laboratory Animal Technology Co., Ltd.). The temperature was maintained at 22°C–24°C in the animal housing, and the humidity was controlled at 50–60%. The animals were subjected to a daily light cycle of 14 h of light and 10 h of darkness. All experimental procedures in this study were approved by the Animal Protection Committee of the Medical School of Peking University (LA2022-001).

### Observation of seizures

Wild-type and *Apoc2*^−/−^ hamsters at 15–17 d postnatal. Pentylenetetrazol (PTZ) powder (Sigma) was dissolved in 0.9% NaCl to prepare a 1% PTZ solution, used immediately. A single-dose seizure induction protocol was used, in which the hamsters were intraperitoneally injected with PTZ solution at a dose of 60 mg/kg body weight to induce seizure activity (23). The seizures were assessed using the Racine seizure severity scale within 30 min after PTZ injection (24). grade 0, no response; grade I, facial clonus (including eye blinking, whisker twitching, rhythmic chewing); grade II, grade I plus nodding movements indicating neck muscle clonus; grade III, grade II plus unilateral forelimb clonus; grade IV, grade III plus rearing and bilateral forelimb clonus; grade V, bilateral forelimb and hindlimb tonic–clonic seizures with body arching and loss of balance, causing the animal to fall. To minimize the impact of hypertriglyceridemia on the absorption of PTZ medication, we made every effort to maintain consistent operations, such as strictly controlling the injection site, depth, and speed of PTZ, and having the same person perform the same operations on hamsters of different groups at a fixed time. We used video monitoring to record the number of grade IV/V seizures in each group of hamsters in real time.

### Whole-cell patch-clamp recordings

Sucrose-based dissecting artificial cerebrospinal fluid (pH 7.3–7.4, osmolarity = 300–310 mOsm): 213 mM sucrose; 3 mM KCl; 0.5 mM CaCl_2_; 5 mM MgCl_2_; 1 mM NaH_2_PO4; 26 mM NaHCO_3_; 10 mM D-glucose; magnesium-free artificial cerebrospinal fluid/perfusate (pH 7.3–7.4, osmolarity = 300–310 mOsm): 124 mM NaCl; 2.5 mM KCl; 2 mM CaCl_2_; 1.25 mM NaH_2_PO4; 26 mM NaHCO_3_; 10 mM D-glucose; intracellular solution (pH 7.3–7.4 adjusted with KOH, osmolarity = 310–320 mOsm): 145 mM KCl; 5 mM NaCl; 5 mM EGTA; 4 mM Mg-ATP; 0.3 mM Na_2_GTP; 10 mM HEPES (all the reagents for the experiments were purchased from Sigma).

Hamsters aged 7–10 d, either wild-type or *Apoc2*^−/−^, were anaesthetized with isoflurane. The heart of each hamster was perfused thoroughly with sucrose-based dissecting solution that was pre-oxygenated with 95% O_2_ and 5% CO_2_ and chilled to an icy slush state. Specifically, the skin is cut under the sternum of the hamster to enter the chest cavity, and the syringe is inserted from the apex of the heart directly into the ascending aorta. A hemostatic forceps is used to clamp the heart to fix the needle tip, and after cutting open the right auricle, the heart is fully perfused (approximately 150 mL of dissection fluid is perfused for each hamster). The brain tissue was removed, and a vibratome (VT1200S, Leica, Germany) was used to obtain coronal brain slices with a thickness of 300 μm. In the slicing chamber, a mixed gas of 95% O_2_ and 5% CO_2_ is continuously flushed in, and the parameters of the vibrating microtome are set to a speed of 0.14 mm/s and a vibration amplitude of 1.0 mm. The slices were transferred to ACSF solution maintained at 32°C for 60 min under continuous oxygenation. After incubation, the brain slices were transferred to a recording chamber and continuously perfused with oxygenated artificial cerebrospinal fluid (at room temperature) at a rate of 3–4 mL/min. Glass microelectrodes (Sutter, America) were pulled into recording glass microelectrodes using an electrode puller (P-97, Sutter, America), and the electrodes were filled with intracellular solution, resulting in an impedance of 4–6 MΩ. Specific layer V pyramidal neurons in the frontal cortex of neonatal hamsters were identified and recorded using a whole-cell patch-clamp amplifier (Axon, America) and recording software. Recording excitatory postsynaptic potentials (EPSPs): In current clamp mode, cells were clamped at 0 pA. Recording evoked action potentials (eAPs): Neurons were given step current stimulation in current clamp mode, depolarizing them stepwise from 0 pA to +300 pA (steps of +20 pA, duration of 500 ms, with intervals of 200 ms). Recording voltage-gated potassium currents: 4 μM 4-AP and 20 μM TEA-Cl were added to block transient outward potassium current (I_A_) and delayed rectifier potassium current (I_K_), respectively. Neurons were stimulated with depolarizing pulses from −80 mV to 100 mV (steps of +10 mV, duration of 200 ms) to record potassium currents.

### Nissl staining and hematoxylin–eosin staining

Hamsters at 15–17 d postnatal were anesthetized with isoflurane and perfused with cold PBS, followed by 4% paraformaldehyde. The brain was removed, soaked in 4% paraformaldehyde for 24 h, and embedded in paraffin. Brain tissues were sectioned at 4 μm using a paraffin microtome and deparaffinized with xylene and alcohol. For Nissl staining, sections were stained with 0.1% toluidine blue for 20 min. For hematoxylin–eosin (HE) staining, sections were treated with xylene and ethanol to remove paraffin, stained with hematoxylin for 5 min, and eosin for 3 min. The stained sections were observed under a microscope (×100) to examine cortical structure.

### Fatty acid profiling of cerebral dialysate

Hamsters were anesthetized with isoflurane and maintained in this state. Each hamster’s head was fixed horizontally in a stereotaxic apparatus (Shenzhen Ruivode, STRONG8003), and a guide cannula was implanted into the right frontal cortex (A: +1 mm, R: +2 mm, V: −2 mm). Body temperature was maintained during and after surgery using an animal temperature maintenance device (Shenzhen Ruivode, 69,001) until recovery. On the second day post-surgery, a microdialysis probe was inserted into the cannula, and a microdialysis system (CMA, Sweden) perfused with compound sodium chloride solution (Beijing Shuanghe Pharmaceutical Co., Ltd.) at 1.5 μL/min to establish a 60 min equilibrium period while hamsters moved freely. Dialysate/brain tissue fluid was then collected for 60 min, yielding approximately 90 μL. An intraperitoneal injection of 1% PTZ at 60 mg/kg was administered, and collection continued for another 60 min. Two dialysate samples (90 μL each) were collected per hamster: one before and one after PTZ injection. Seizure occurrence was monitored during collection.

Free fatty acids (FFAs) were extracted from the collected brain tissue fluid using a modified Bligh and Dyer method (25). The analysis of free fatty acids was performed using a Shimadzu Nexera 20 AD-HPLC/ExionLC-AD triple quadrupole/ion trap mass spectrometer (6,500 Plus QTRAP; SCIEX) (26). Additionally, short-chain fatty acids were extracted using acetonitrile, then derivatized and analyzed using a gas chromatography machine (27).

### Transcriptomic analysis

Hamsters at 15–17 postnatal were selected, and the seizure model was established as previously described. Following successful model induction, cortical brain tissue from each group of hamsters was carefully harvested for comprehensive transcriptomic analysis to capture relevant neuronal alterations. The Illumina mRNA TruSeq Kit was employed for library construction, followed by stringent quality control and high-throughput sequencing. This approach facilitated the identification and comparative analysis of differentially expressed genes, thereby offering a robust foundation for elucidating the underlying molecular mechanisms.

### RNA extraction and quantitative real-time PCR

Total RNA from cortex of hamsters was extracted with TRIzol reagent (Invitrogen, United States), and the first-strand cDNA was generated with a reverse transcription (RT) kit (Promega, United States). Quantitative real-time PCR (RT-qPCR) was conducted using an Applied Biosystems with BRYT Green fluorescence (Promega, United States). The target mRNA levels were normalized to GAPDH internal control gene. ZDHHC14 and GAPDH were amplified by PCR using 5’-CAGAGTGACATGTGCGACCA-3′ and 5’-CCCAGGCATATGCAGCTCTT-3′, 5’-GACTCATGACCACAGTCCATGC-3′ and 5’-AGAGGCAGGGATGATGTTCTG-3′ as primers, respectively.

### Statistical analysis

All data in the manuscript are presented as the mean ± standard error of the mean (SEM). *n* represents the number of pyramidal neurons in layer V of the frontal cortex, unless otherwise specified. EPSPs were analyzed using Mini Analysis 6.0.3 software, and action potentials were analyzed using Clampfit 10.1 software. The data in the manuscript follow a normal distribution, and paired or unpaired Student’s *t* tests were performed, including comparisons of action potential frequency and amplitude between two groups of hamsters under the same current stimulation, as well as comparisons of potassium ion current magnitude between two groups of hamsters under the same voltage stimulation. When comparing the same indicator between two groups, we used the unpaired *t*-test; when comparing indicators before and after drug administration, we used the paired *t*-test for analysis. *p < 0.05, **p < 0.01 and ***p < 0.001 indicate statistical significance.

## Results

### *Apoc2*^−/−^ hamsters exhibited fewer grade IV/V seizures than wild-type hamsters following PTZ induction

**Figure 1:**
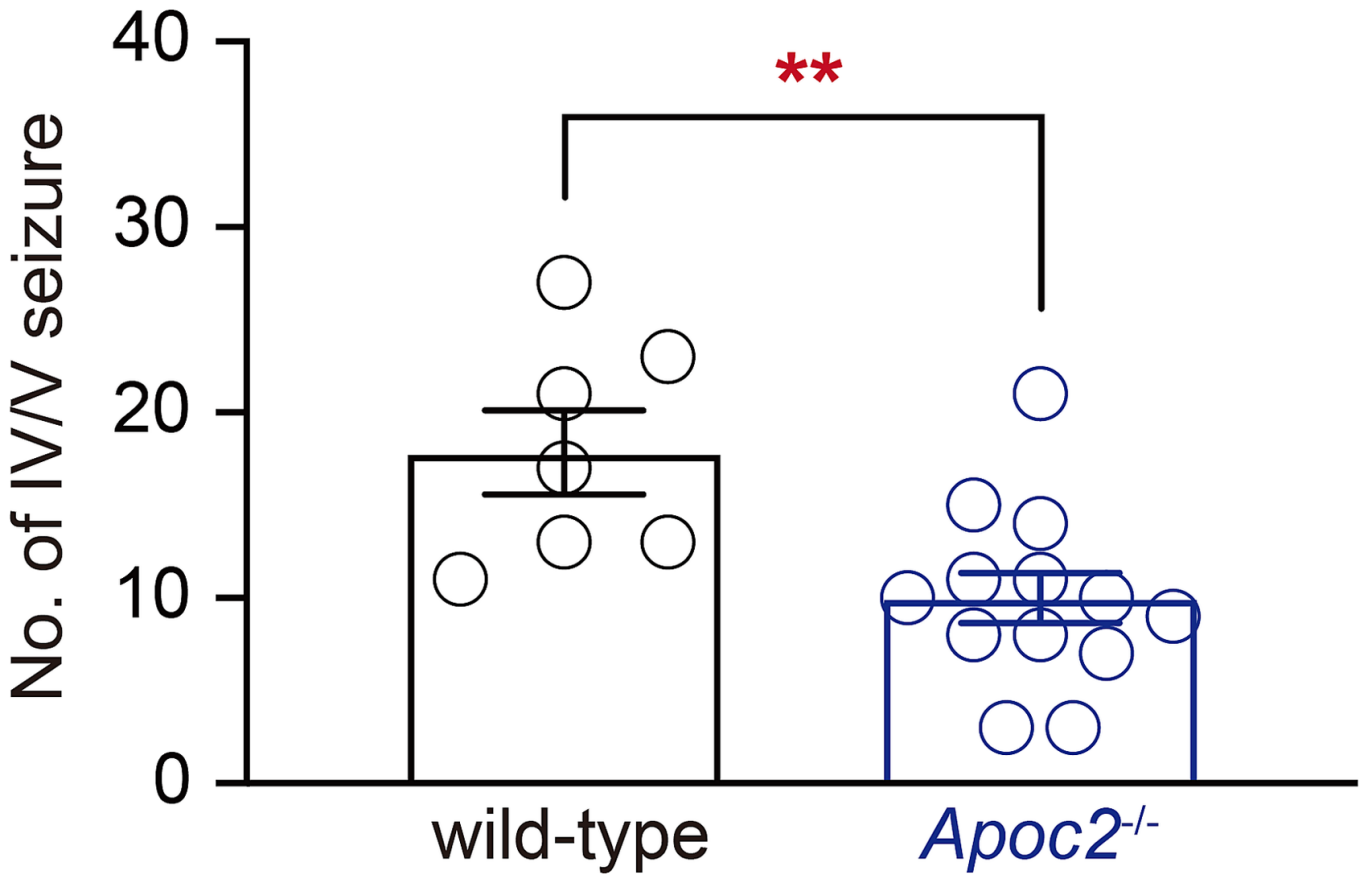
Statistics on the frequency of seizures in hamsters following PTZ induction. After seizure induction via intraperitoneal administration of PTZ, *Apoc2*^−/−^ hamsters exhibited a significantly lower frequency of IV/V-grade seizure episodes compared to wild-type hamsters.

Seizures were induced in hamsters using a single dose of PTZ administered intraperitoneally. The results demonstrated that the frequency of grade IV/V seizures recorded within 30 min post-PTZ administration was significantly lower in the *Apoc2*^−/−^ group compared to the wild-type group (shown in Figure 1 wild-type group: 17.86 ± 2.26 times, *n* = 7; *Apoc2*^−/−^ group: 10.00 ± 1.34 times, *n* = 13; *p* < 0.01). *n* represents the number of hamsters recorded in each group.

### The excitability of pyramidal neurons in the frontal cortex of *Apoc2*^−/−^ hamsters was lower than that of wild-type hamsters

**Figure 2:**
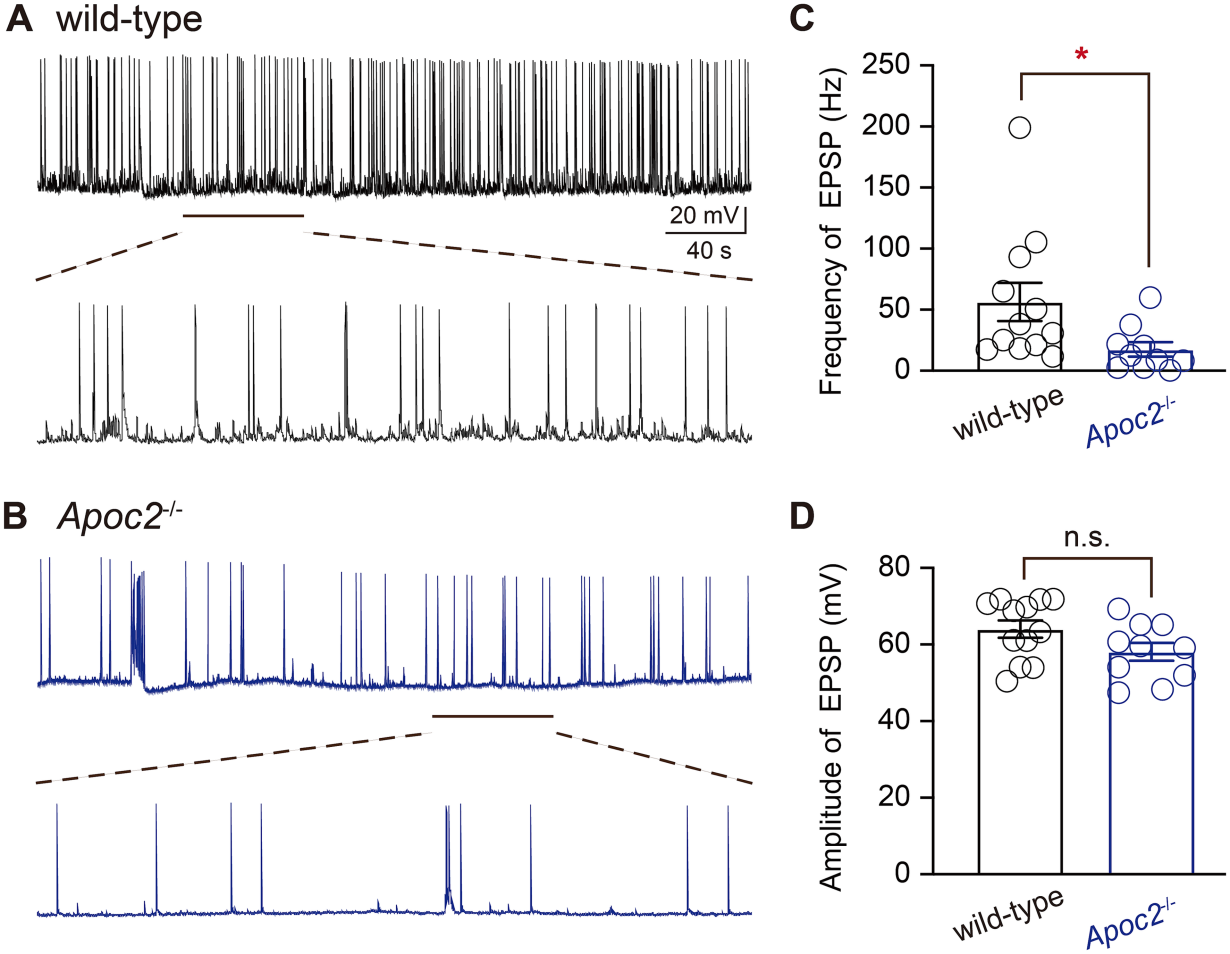
Schematic diagram of excitatory postsynaptic potentials (EPSPs) in pyramidal neurons of the hamster frontal cortex, along with frequency and amplitude statistics. **(A)** Top: EPSPs recorded from wild-type hamsters; bottom: magnified view of EPSPs recorded from wild-type hamsters (1 min). **(B)** Top: EPSPs recorded from *Apoc2*^−/−^ hamsters; bottom: magnified view of EPSPs recorded from *Apoc2*^−/−^ hamsters (1 min). **(C)** Frequency distribution of EPSPs in both groups, demonstrating a significant reduction in EPSP frequency in *Apoc2*^−/−^ hamsters compared to wild-type hamsters. **(D)** Amplitude distribution of EPSPs in both groups, indicating no significant difference in EPSP amplitudes between *Apoc2*^−/−^ hamsters and wild-type hamsters.

Whole-cell patch-clamp recording techniques were employed to compare the excitability of pyramidal neurons in layer V of the frontal cortex between wild-type and *Apoc2*^−/−^ hamsters by recording EPSPs (Figures 2A,B). Experimental results demonstrated that the frequency of EPSPs recorded within 6 min for pyramidal neurons in layer V of the frontal cortex was significantly lower in the *Apoc2*^−/−^ group of hamsters than in the wild-type group (shown in Figure 2C, wild-type group: 56.31 ± 15.67 Hz, *n* = 12 from 4 hamsters; *Apoc2*^−/−^ group: 17.47 ± 5.92 Hz, *n* = 10 from 4 hamsters; *p* < 0.05). No significant difference was observed in the amplitude of EPSPs between the two groups (shown in Figure 2D, wild-type group: 64.05 ± 2.28 mV; *Apoc2*^−/−^ group: 58.10 ± 2.36 mV; *p* = 0.09).

**Figure 3:**
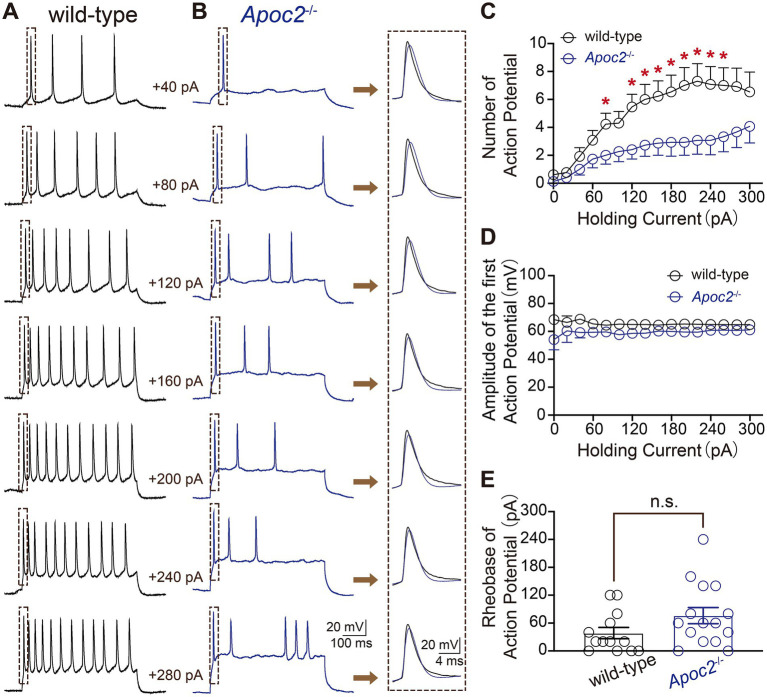
Schematic diagram of evoked action potentials (eAPs) in pyramidal neurons of the hamster frontal cortex, along with frequency and amplitude statistics. **(A)** eAPs recorded under various current stimulations in wild-type hamsters, with each small green dashed box indicating the first eAP elicited under different current stimulations. **(B)** eAPs recorded under various current stimulations in *Apoc2*^−/−^ hamsters, with each small green dashed box indicating the first eAP elicited under different current stimulations. **(C)** Statistics on the number of eAPs elicited under different current stimulations in the two hamster groups. *Apoc2*^−/−^ hamsters exhibited a significant decrease in the number of eAPs elicited under +80 pA and + 120 pA to +260 pA current stimulations compared to wild-type hamsters. **(D)** Statistics on the amplitude of the first elicited action potential under different current stimulations in the two groups, demonstrating no significant difference between the groups. **(E)** Statistics on the minimum current stimulation required to evoke action potential firing (threshold current stimulation) in the two groups, showing no significant difference between the threshold current stimulations of the groups.

Additionally, eAPs were recorded from pyramidal neurons in layer V of the frontal cortex in both groups of hamsters to compare neuronal excitability. The experiment revealed that at different levels of current stimulation, the frequency of action potentials in neurons from the *Apoc2*^−/−^ group of hamsters was significantly lower than that in neurons from the wild-type group of hamsters (shown in Figure 3; Supplementary Table S1). The action potential frequency in *Apoc2*^−/−^ hamster neurons was markedly lower than that in wild-type hamster neurons at current stimulations of +80 pA, +120 pA, and + 260 pA. However, no significant difference was observed in the amplitude of the first action potential between the two groups under different current stimulations (shown in Figure 3D). Furthermore, the threshold current stimulation required for action potential generation in pyramidal neurons of the frontal cortex was higher in the *Apoc2*^−/−^ group of hamsters than in the wild-type group (shown in Figure 3E, wild-type group: 38.46 ± 12.08 pA, *n* = 13 from 4 hamsters; *Apoc2*^−/−^ group: 76.00 ± 16.37 pA, *n* = 15 from 5 hamsters; *p* = 0.10), but the difference was not statistically significant.

The aforementioned results indicate that the excitability of pyramidal neurons in layer V of the frontal cortex is significantly lower in *Apoc2*^−/−^ hamsters than in wild-type hamsters.

### No significant differences were observed in the cortical morphology between *Apoc2*^−/−^hamsters and wild-type hamsters

**Figure 4:**
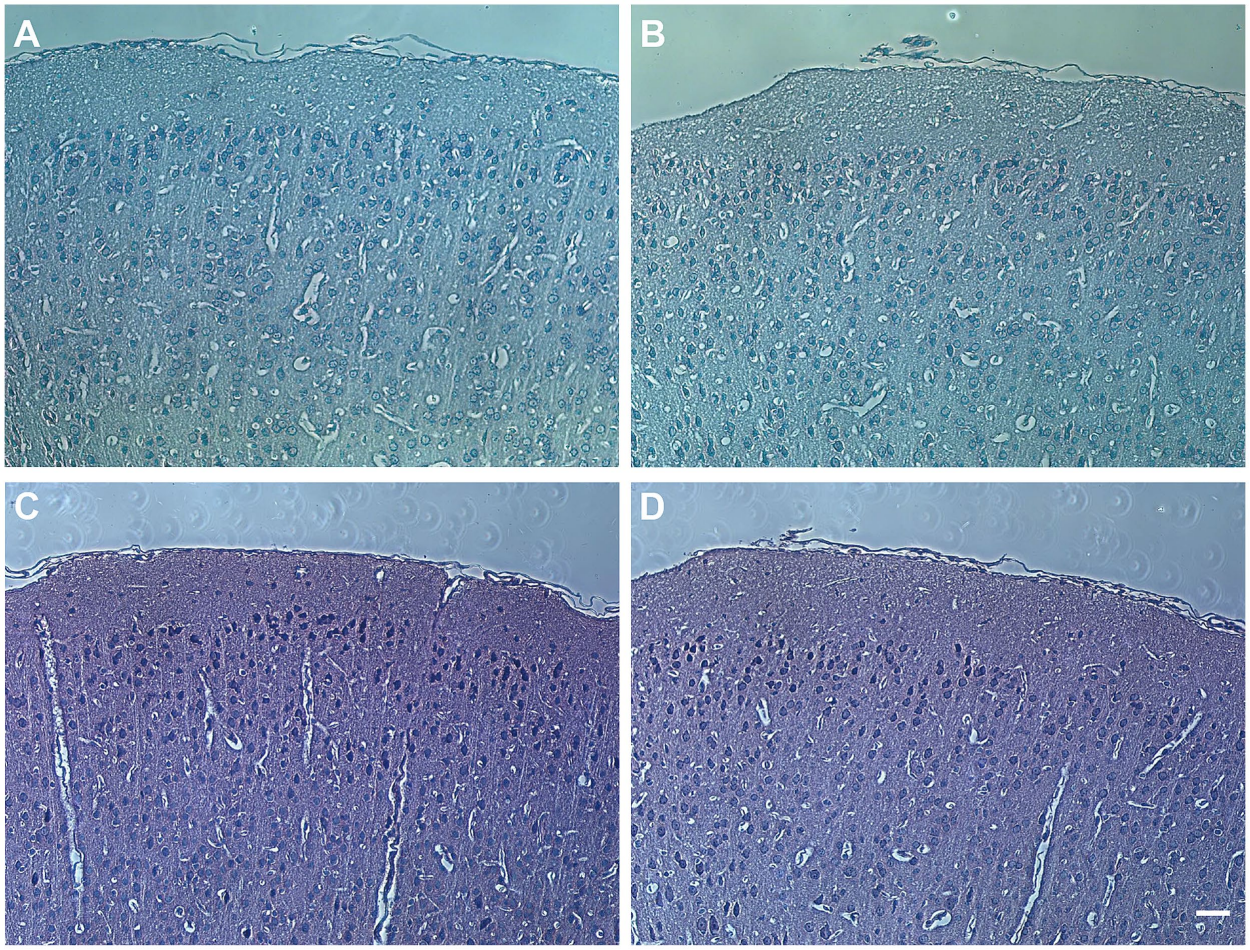
Hamster frontal cortex brain sections stained with hematoxylin and eosin (HE) and Nissl staining. **(A)** Pathological image of wild-type hamster brain with HE staining. **(B)** Pathological image of *Apoc2*^−/−^ hamster brain with HE staining. **(C)** Pathological image of wild-type hamster brain with Nissl staining. **(D)** Pathological image of *Apoc2*^−/−^ hamster brain with Nissl staining. Scale bar = 50 μm.

Following PTZ injection, the morphology of the frontal cortex in wild-type and *Apoc2*^−/−^ hamsters was assessed using HE and Nissl staining. In both groups, the cortex appeared structurally intact with normal neuronal morphology and dense neuronal arrangement. These findings indicate that elevated triglyceride levels did not lead to neuronal loss or alter the morphology of the frontal cortex in infantile hamsters (shown in Figure 4). Therefore, *Apoc2*^−/−^ hamsters demonstrate increased resistance to seizures and reduced neuronal excitability, with these effects not being attributable to any structural damage within the frontal cortex.

### The concentration of free palmitic acid in the frontal cortex dialysate was lower in *Apoc2*^−/−^ hamsters than in the wild-type group

**Figure 5:**
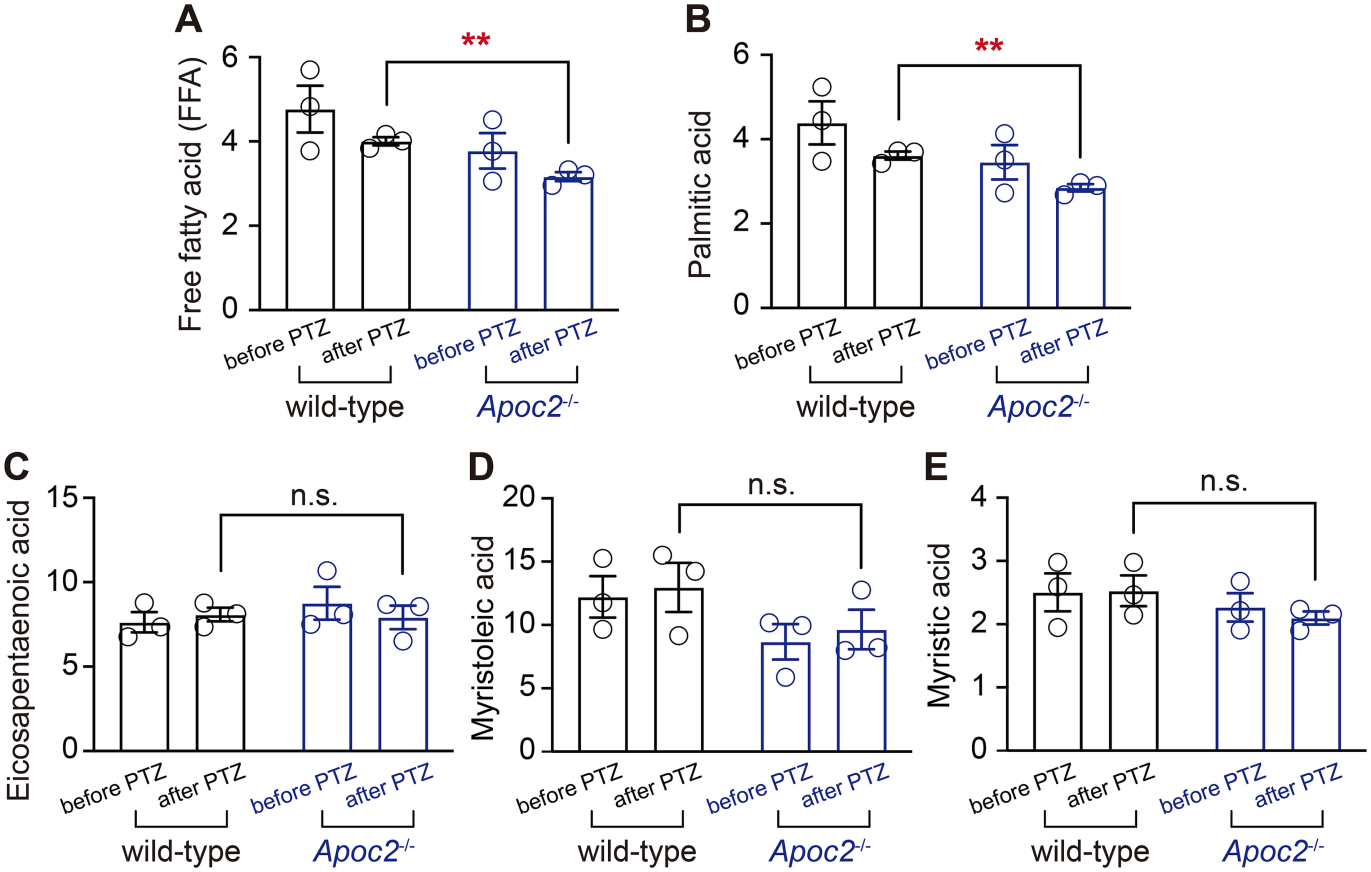
Statistical graph of long-chain free fatty acid content in the frontal cortex dialysate of hamsters. Following PTZ-induced seizures, a downward trend was observed in the levels of long-chain free palmitic acid in both groups. *Apoc2*^−/−^ hamsters exhibited a significant reduction in the content of long-chain free palmitic acid among total free fatty acids (FFA, **A**) and palmitic acid **(B)** compared to wild-type hamsters. In contrast, no significant differences were observed in eicosapentaenoic acid **(C)**, myristoleic acid **(D)**, and myristic acid **(E)**.

To investigate the lipid molecules involved in the seizure-protective effect of *Apoc2*^−/−^ hypertriglyceridemic hamsters, we focused on the extracellular fluid, which connects neurons with the peripheral lipid environment (Figure 5A). Free fatty acid lipidomics were performed on the frontal cortex dialysate of wild-type and *Apoc2*^−/−^ hamsters after PTZ-induced seizures. The results indicated a significant reduction in palmitic acid in the frontal cortex microdialysate of the *Apoc2*^−/−^ hamster group after seizure induction (shown in Figure 5B, wild-type group: 36.15 ± 0.95 nmol/L, *n* = 3; *Apoc2*^−/−^ group: 29.00 ± 0.87 nmol/L, *n* = 3; *p* < 0.01). Additionally, free palmitic acid in the frontal cortex of both groups was lower post-seizure compared to pre-seizure levels. There were no statistically significant differences in the levels of other long-chain fatty acids such as eicosapentaenoic acid, myristoleic acid, and myristic acid (shown in Figures 5C–E). We also examined the changes in the content of medium-chain and short-chain fatty acids and found no significant differences between the two groups of hamsters (shown in Supplementary Figure S1).

### The expression of palmitoyl acyltransferase ZDHHC14 in the frontal cortex tissue was higher in *Apoc2*^−/−^ hamsters than in the wild-type group

**Figure 6:**
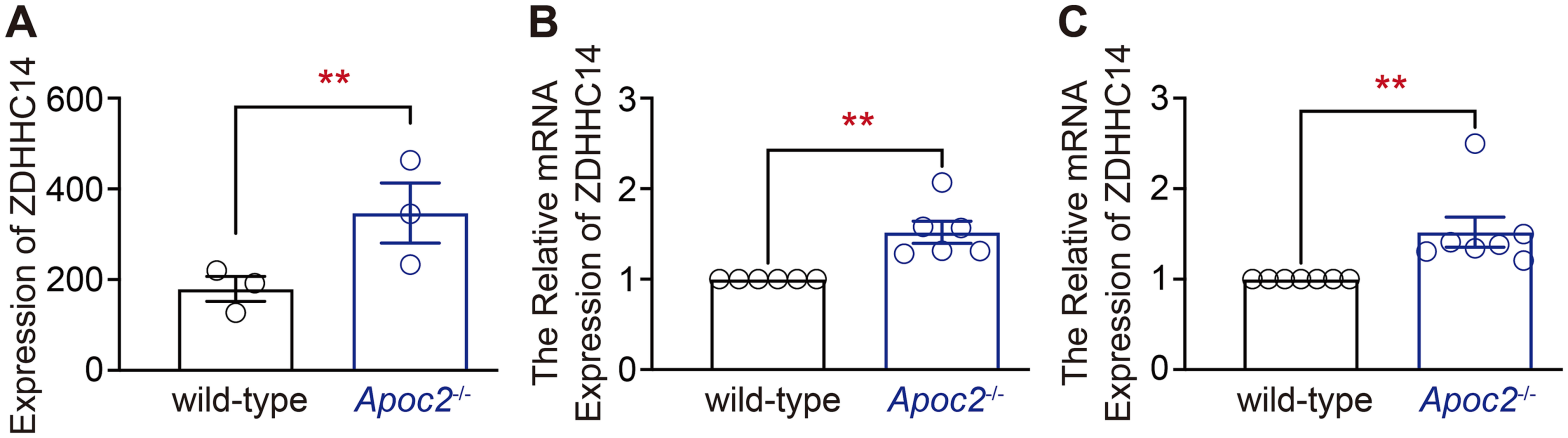
Statistical graph of palmitoyl acyltransferase ZDHHC14 expression in the hamster frontal cortex. Expression of ZDHHC14 in the frontal cortex of *Apoc2*^−/−^ hamsters was elevated relative to wild-type hamsters both prior to and following seizure induction. **(A)** Transcriptome sequencing results of the frontal cortex after PTZ-induced seizures. **(B)** Quantitative analysis of ZDHHC14 mRNA expression relative to GAPDH in hamsters subjected to PTZ injection. **(C)** Quantitative analysis of ZDHHC14 mRNA expression relative to GAPDH in hamsters not subjected to PTZ injection.

Given the experimental results indicating a significant reduction in free palmitic acid in the frontal cortex of *Apoc2*^−/−^ hamsters after seizure induction, we hypothesized that genes related to free palmitic acid metabolism might be differentially expressed in the frontal cortex of the two groups of hamsters. Therefore, we screened for genes associated with palmitic acid in the differentially expressed gene database derived from the transcriptomic data. We found that palmitoyl acyltransferase ZDHHC14 was significantly upregulated in the cortex of *Apoc2*^−/−^ hamsters (shown in Figure 6A wild-type group: 179.67 ± 27.55, *n* = 3; *Apoc2*^−/−^ group: 347.00 ± 66.40, *n* = 3; *p* = 0.08). Further validation by RT-PCR demonstrated that the expression of ZDHHC14 in the frontal cortex of *Apoc2*^−/−^ hamsters was significantly elevated compared to wild-type hamsters both before (Figure 6C) and after seizure induction (shown in Figure 6B). *n* represents the number of hamsters recorded in each group.

### The transient outward potassium current in the frontal cortex neurons was enhanced in *Apoc2*^−/−^ hamsters compared to the wild-type group

In hippocampal neurons, ZDHHC14 regulates the strength of voltage-gated potassium currents by mediating the palmitoylation of the scaffold protein PSD93 (28). To investigate whether the upregulation of ZDHHC14 in the frontal cortex of *Apoc2*^−/−^ hamsters also influences the magnitude of voltage-gated potassium currents, we examined two major subtypes of these currents, transient outward potassium current (I_A_) and delayed rectifier potassium current (I_K_), in an *ex vivo* seizure model.

**Figure 7:**
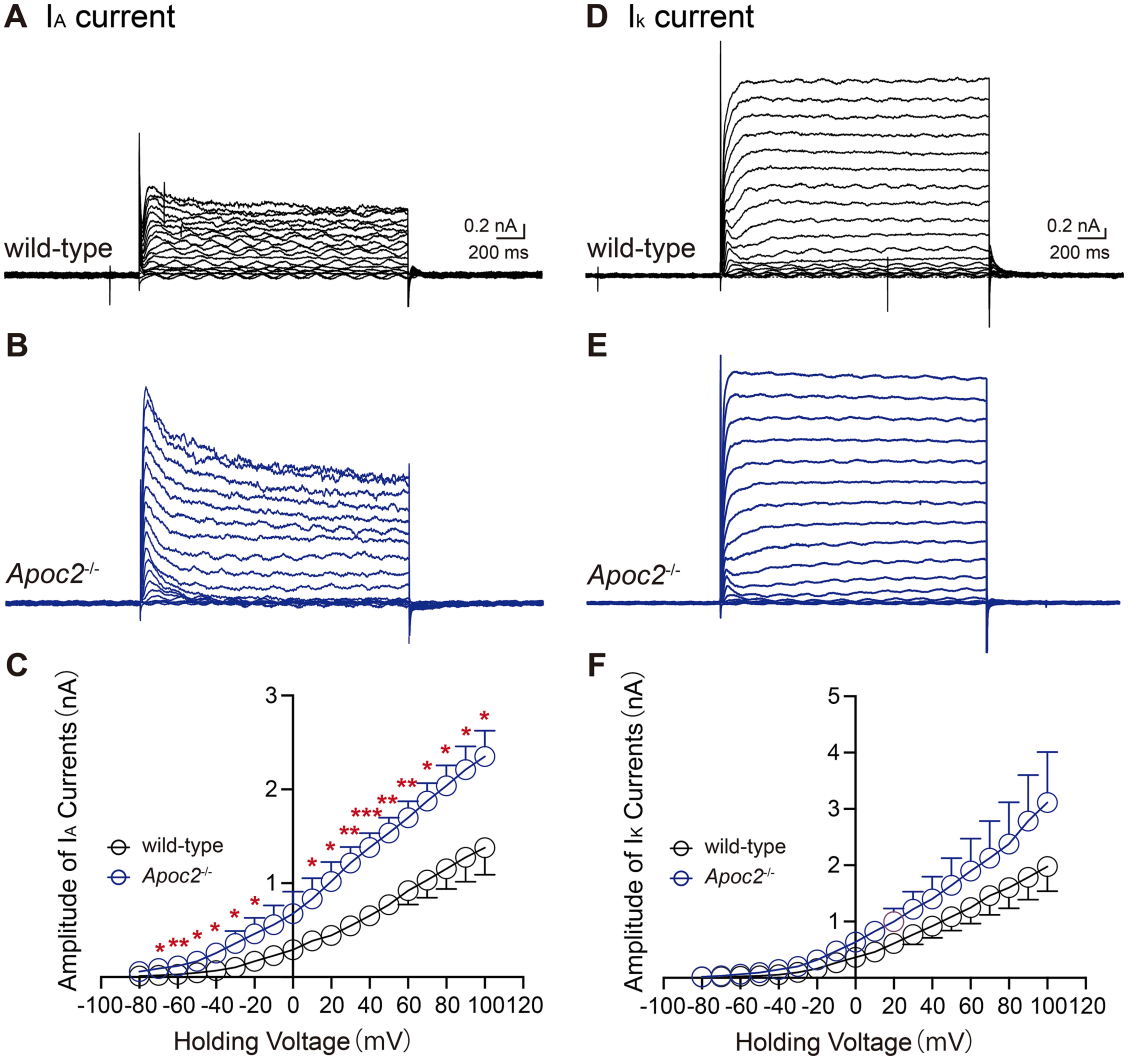
Schematic diagram and current amplitude statistics of the transient outward potassium current (I_A_) and the delayed rectifier potassium current (I_K_) in pyramidal neurons of the hamster frontal cortex. *Apoc2*^−/−^ hamsters showed a significant increase in I_A_ current amplitude, while I_DR_ amplitude remained unchanged. **(A)** I_A_ recorded from the wild-type group under different voltage stimulations. **(B)** I_A_ recorded from the *Apoc2*^−/−^ group under different voltage stimulations. **(C)** Statistical analysis of I_A_ current amplitudes in both groups across varying voltage stimulations. **(D)** I_K_ recorded from the wild-type group under different voltage stimulations. **(E)** I_K_ recorded from the *Apoc2*^−/−^ group under different voltage stimulations. **(F)** Statistical analysis of I_K_ current amplitudes in both groups across varying voltage stimulations.

We compared I_A_ and I_K_ in the frontal cortex neurons of both groups of hamsters. Statistical analysis of the current amplitudes induced by a series of step depolarizations from −80 mV to +100 mV revealed that the I_A_ current amplitude in the frontal cortex neurons of *Apoc2*^−/−^ hamsters was significantly enhanced compared to that in wild-type hamsters under multiple depolarizing voltage stimuli (shown in Figures 7A–C; Supplementary Table S2). Measurement of potassium ion currents in frontal cortex neurons showed that, compared to wild-type hamsters, *Apoc2*^−/−^ hamsters exhibited significantly increased amplitude of I_A_ (−80 mV, wild-type group: 0.01 ± 0.00 nA, *n* = 8 from 4 hamsters; *Apoc2*^−/−^ group: 0.06 ± 0.02 nA, *n* = 4 from 3 hamsters; *p* < 0.05. +30 mV, wild-type group: 0.55 ± 0.08 nA; *Apoc2*^−/−^ group: 1.22 ± 0.17 nA; *p* < 0.01. +100 mV, wild-type group: 1.36 ± 0.28 nA; *Apoc2*^−/−^ group: 2.35 ± 0.28 nA; *p* < 0.05). However, upon conducting a meticulous comparison of the I_K_ amplitude within the frontal cortex neurons between *Apoc2*^−/−^ and wild-type hamsters, it was observed that there were no statistically significant differences present (shown in Figures 7D–F; Supplementary Table S3).

## Discussion

Neonates have the highest incidence of seizures among all age groups. The effectiveness rate of anti-seizure medication is only approximately half, and if the condition is not well controlled, it can result in residual neurological sequelae. Triglycerides—the primary fat source in the ketogenic diet—have an unclear role. Studying the role of high triglycerides in seizures can provide new insights for identifying novel therapeutic targets. We chose an *Apoc2*^−/−^ hypertriglyceridemic hamster model and compared it with a control group. The HTG hamsters exhibited a reduction in the frequency of acute seizure attacks. *Ex vivo* studies also demonstrated a decrease in neuronal excitability, indicating that HTG indeed has a protective effect when there is excessive synchronous neuronal firing. Furthermore, the reduction in free palmitic acid content and the increased expression of ZDHHC14 suggest that palmitoylation modification may be a mechanism underlying its protective effect.

First, the *in vivo* results showed significantly fewer tonic–clonic seizures in *Apoc2*^−/−^ hamsters than in wild-type hamsters. Given the size of hamsters aged P7–P10 is relatively small, we chose P15–P17 to establish an acute seizure model. Hypertriglyceridemia may also introduce confounding factors in terms of the efficiency of drug absorption via intraperitoneal injection. Therefore, we also conducted *ex vivo* experiments using brain slices from neonatal hamsters aged P7–P10, inducing neuronal discharges by perfusing brain slices with magnesium-free artificial cerebrospinal fluid. The results indicated that the EPSP frequency of cortical neurons in HTG hamsters was significantly lower than that in wild-type hamsters, and the AP frequency was also significantly lower under various intensities of electrical stimulation. This suggests that cortical neurons in HTG hamsters exhibit lower excitability than those in wild-type hamsters, which is consistent with the results of *in vivo* experiments. Consequently, we have confirmed at both *in vivo* and *ex vivo* levels that hypertriglyceridemia reduces neuronal excitability in the seizure model and has an inhibitory effect on the synchronization of the firing of a large number of neurons.

To our knowledge, no studies have investigated the effect of hypertriglyceridemia on acute seizures, particularly on neuronal excitability in neonatal seizure models. One possible mechanism underlying the anti-seizure effect of a ketogenic diet is that it provides medium-chain fatty acids and ketone bodies as alternative energy sources for epileptogenic brain regions (29, 30). Therefore, the lipid environment of hypertriglyceridemia may provide abundant metabolic substrates for the nervous system, meeting the energy demands of synchronized neuronal firing. To further investigate the mechanisms, we focused on the extracellular fluid of the cortex, which serves as a mediator between the nervous system and the periphery. We collected microdialysates from *Apoc2*^−/−^ and wild-type hamsters before and after seizures and conducted fatty acid metabolomics. We found a trend towards a decrease in free palmitic acid in both wild-type and *Apoc2*^−/−^ hamsters after seizure induction compared to pre-seizure levels, with a significant reduction in free palmitic acid in *Apoc2*^−/−^ hamsters compared to wild-type post-seizure. This finding aligns with prior research by Leitner et al., which demonstrated a reduction in cortical palmitic acid levels in epilepsy patients on a ketogenic diet, suggesting a possible association between reduced free palmitic acid and seizure protection (31). However, the specific physiological role of this reduction in modulating neuronal excitability remains to be elucidated. The neuroprotective roles of fatty acids remain a significant research focus. While our study centers on palmitic acid and no significant differences were observed in the levels of other fatty acids, it is possible that other fatty acids and lipid metabolites, such as caprylic acid, capric acid, oleic acid, linoleic acid, and polyunsaturated fatty acids, may also contribute through mechanisms like *α*-amino-3-hydroxy-5-methyl-4-isoxazolepropionic acid (AMPA) receptor inhibition, PPAR-*γ* activation, and anti-inflammatory or antioxidant effects (32–34). Given the age-specific impacts of fatty acids on neuronal protection, systematic analyses of their metabolic profiles and functions in neonatal seizure models could advance the development of personalized therapeutic strategies for neonatal seizures.

Cortical transcriptomics and qPCR showed an upregulation of ZDHHC14 expression in the cortex of *Apoc2*^−/−^ hamsters before and after PTZ injection. Since free palmitic acid serves as the substrate for protein palmitoylation, these findings suggest an increased level of palmitoylation in the cortex of *Apoc2*^−/−^ HTG hamsters. Palmitoylation, the covalent attachment of a palmitic acid ester to cysteine residues in proteins, is one of the most important lipid modifications. Protein palmitoylation dynamically regulates the transport of proteins between the plasma membrane and intracellular compartments such as the Golgi apparatus, endoplasmic reticulum, and endocytic vesicles (35, 36). In the nervous system, palmitoylation is closely associated with the transport of ion channel receptors and ion channels. Kang et al. published a study in Nature that conducted a palmitoyl-proteomics analysis on rat cortical neurons, identifying 89 palmitoylated proteins, including neurotransmitter receptors, ion channels, and scaffold proteins. Most of these proteins are transmembrane proteins, with palmitoylation contributing to their targeted trafficking to membrane microdomains. Furthermore, after inducing seizures in the animals, Kang et al. re-examined the levels of protein palmitoylation in neurons and found an increase in palmitoylation levels for most proteins (37). Spinelli et al. found that overexpression of ZDHHC3 can lead to enhanced palmitoylation modification of the GluA1 subunit of AMPA receptors, inhibit their stimulus-dependent trafficking to the plasma membrane, and thereby affect synaptic plasticity (38). Mice with palmitoylation-deficient C-terminal GluA1 subunits of AMPA receptors exhibit increased brain hyperexcitability and heightened seizure susceptibility (39). The palmitoylation modification of Kv1.5 can regulate its stable expression on the membrane (40).

Palmitoyl acyltransferases (PATs), which contain a DHHC (Asp-His-His-Cys) zinc finger domain, are responsible for catalyzing palmitoylation modification reactions. Therefore, they are also known as the ZDHHC protein family. In mammals, 23 members of this family have been identified (41, 42). The functions of each member of the ZDHHC family are still being intensively researched. Although existing evidence suggests that protein palmitoylation is crucial for maintaining the normal physiological functions of neuronal ion channels and ion channel receptors (40, 43–45), research on the role of protein palmitoylation in the onset and progression of epilepsy remains insufficient. Kang et al. found that after inducing seizures in rats, the level of neuronal protein palmitoylation was enhanced compared to the non-convulsive group (37), indicating that the regulation of palmitoylation is closely related to neuronal firing activity. It warrants further investigation to uncover novel therapeutic targets.

Our results suggest that ZDHHC14 expression in the cerebral cortex of HTG hamsters is upregulated after seizure induction. Although ZDHHC14 is highly expressed in the brain, there is limited research on its functions in the nervous system. A recent study by Sanders et al. suggests that ZDHHC14 in the hippocampus mediates palmitoylation of postsynaptic density 93 and type I voltage-gated potassium (Kv1) channels, targeting them to the axon initial segment (AIS). Moreover, the loss of ZDHHC14 leads to reduced palmitoylation of Kv1 channels, resulting in a decrease in AIS-targeted potassium channels and a subsequent reduction in voltage-dependent outward currents, thereby, increasing neuronal excitability (28). To date, there has been no functional research on ZDHHC14 in the cortex. Therefore, we further examined the magnitude of voltage-gated potassium currents in the cortical neurons. The results showed that the transient outward potassium current (I_A_) was enhanced in the cortical neurons of *Apoc2*^−/−^ hamsters. I_A_ can slow the approach of the membrane potential to the threshold in excited neurons, prolong the inter-spike interval of action potentials, and reduce the firing frequency of action potentials (46, 47). Additionally, I_A_ can quickly recover from inactivation, facilitating the repolarization of action potentials. This is consistent with our observation of decreased excitatory postsynaptic potentials and evoked action potential frequencies in the frontal cortex neurons of *Apoc2*^−/−^ hamsters.

This study revealed the differential expression of ZDHHC14, highlighting its significant role in regulating neuronal excitability and conferring protection against seizures. However, given the complexity of the ZDHHC family and the potential functional redundancy among its members, other ZDHHC proteins may also contribute to this process. For instance, ZDHHC17 has been shown to catalyze the palmitoylation of the voltage-gated potassium channel Kv1.1, thereby regulating its subcellular localization at the axon initial segment (43). Similarly, ZDHHC3 and ZDHHC15 have been demonstrated to influence synaptic transmission and plasticity through their regulatory roles in the trafficking of AMPA receptors, NMDA receptors, and GABA_A_ receptors (45, 48–50). Moreover, the crystal structures of ZDHHC family members remain insufficiently characterized, which may account for substrate overlap among some members. In this study, while differential expression of ZDHHC14 was observed, the expression levels and functions of other family members have yet to be comprehensively evaluated. Therefore, future studies will employ gene knockout or overexpression approaches to further elucidate the specific role of ZDHHC14 in neuronal excitability and seizure modulation.

In recent years, a few studies have begun to focus on the impact of high triglycerides on palmitoylation. Our research results also suggest a potential association between hypertriglyceridemia and palmitoylation. However, we are currently unable to provide a detailed explanation for this, and further research is needed. Spinelli et al. found that a high-fat diet induced insulin resistance in the hippocampus of mice, leading to upregulated expression of FOXO3a-mediated ZDHHC3 and increased palmitoylation levels in the hippocampus (38). It is evident that the lipid abundance provided by high triglycerides offers substrates for palmitoylation in the nervous system, maintaining active palmitoylation activity and resulting in more pronounced molecular interactions. However, in the *Apoc2*^−/−^ HTG hamster cortex used in this study, no changes were observed in the expression of insulin resistance-related genes. The outcomes of neurological experiments depend heavily on the age of the animals. In Spinelli’s experiment, adult mice aged 10–11 weeks were selected, with the research focus on synaptic plasticity of hippocampal neurons rather than excessive abnormal synchronization of numerous cortical neurons, which differs from the focus of this study.

The ketogenic diet, which utilizes fat as the primary energy source, has demonstrated well-established anti-seizure effects. However, its application in the neonatal period is substantially constrained due to the requirement for high-fat intake, which can result in feeding intolerance, hypoglycemia, and severe metabolic acidosis, potentially posing life-threatening risks (51). Elucidating the mechanisms by which triglycerides regulate neuronal excitability, including the identification of specific pathways and molecular targets, holds promise for optimizing ketogenic therapies and developing precision-targeted pharmacological interventions, such as ZDHHC14 modulators, for use in conjunction with lipid-based therapies like the ketogenic diet. Despite the potential of voltage-gated potassium channels as therapeutic targets for anti-seizure drug development, relatively few pharmacological agents specifically target these channels. The role of ZDHHC14-mediated palmitoylation in regulating voltage-gated potassium channels remains incompletely understood. This highlights a significant avenue for clinical research. Investigating the catalytic domains and regulatory regions of ZDHHC14, alongside resolving its three-dimensional crystal structure, could facilitate the development of novel therapeutic strategies for epilepsy management.

This study identified a potential regulatory role of ZDHHC14 in the cortex on transient outward potassium current. However, further research is required to elucidate how ZDHHC14 influences transient outward potassium channels in the cortex, its regulatory effects on the intracellular trafficking of potassium channels, and the specific subtypes of interacting target proteins. These investigations are essential to clarify the functional role of ZDHHC14 in the nervous system and its mechanisms for modulating neuronal excitability.

In summary, this study demonstrates that the *Apoc2*^−/−^ hypertriglyceridemic hamster model exhibits a reduced frequency of induced seizures and decreased cortical neuron excitability. These findings confirm, both *in vivo* and *ex vivo*, the seizure-protective effects of elevated triglycerides. Mechanistic exploration indicates that the upregulation of ZDHHC14 expression in the cortex, leading to enhanced transient outward potassium currents, may contribute to this protective effect. This study provides novel insights into potential therapeutic targets for neonatal seizures. Future research will focus on further elucidating the role of ZDHHC14 in modulating neuronal excitability in the cortex by knocking down or overexpressing ZDHHC14. Additionally, we will explore the relationship between hypertriglyceridemia and neural palmitoylation.

## Data Availability

The raw data supporting the conclusions of this article will be made available by the authors, without undue reservation.

## Supplementary material

SUPPLEMENTARY FIGURE S1 Statistical graph of short- and medium-chain free fatty acid content in the frontal cortex dialysate of hamsters. Following PTZ-induced seizures, there was no statistically difference in the long-chain free fatty acid levels of both the wild-type hamsters and *Apoc2*^−/−^ hamsters, including total short- **(A)**, medium-chain free fatty acid **(B)**, acetic acid **(C)**, propionic acid **(D)**, butyric acid **(E)**, isobutyric acid **(F)**, 2-methyl-butyric acid **(G)**, isovaleric acid **(H)**, valeric acid **(I)**, isocapric acid **(J)**, capric acid **(K)**, heptanoic acid **(L)**, octanoic acid **(M)**, nonanoic acid **(N)**, decanoic acid **(O)** and lauric acid **(P)**. *n*=3, *n* represents the number of hamsters recorded in each group. SCFA, short-chain free fatty acid; MCFA, medium-chain free fatty acid.
