# Supplementary material for: Decreased neuronal excitability in hypertriglyceridemia hamsters with acute seizures
Source: Front Neurol. 2024 Dec 19;15:1500737. doi: 10.3389/fneur.2024.1500737 (PMC11730077; doi:10.3389/fneur.2024.1500737)

**Supplementary Figure S1** Statistical graph of short- and medium-chain free fatty acid content in the frontal cortex dialysate of hamsters. Following PTZ-induced seizures, there was no statistically difference in the long-chain free fatty acid levels of both the wild-type hamsters and *Apoc2*^-/-^ hamsters, including total short- (**A**), medium-chain free fatty acid (**B**), acetic acid (**C**), propionic acid (**D**), butyric acid (**E**), isobutyric acid (**F**), 2-methyl-butyric acid (**G**), isovaleric acid (**H**), valeric acid (**I**), isocapric acid (**J**), capric acid (**K**), heptanoic acid (**L**), octanoic acid (**M**), nonanoic acid (**N**), decanoic acid (**O**) and lauric acid (**P**). SCFA: short-chain free fatty acid; MCFA: medium-chain free fatty acid.


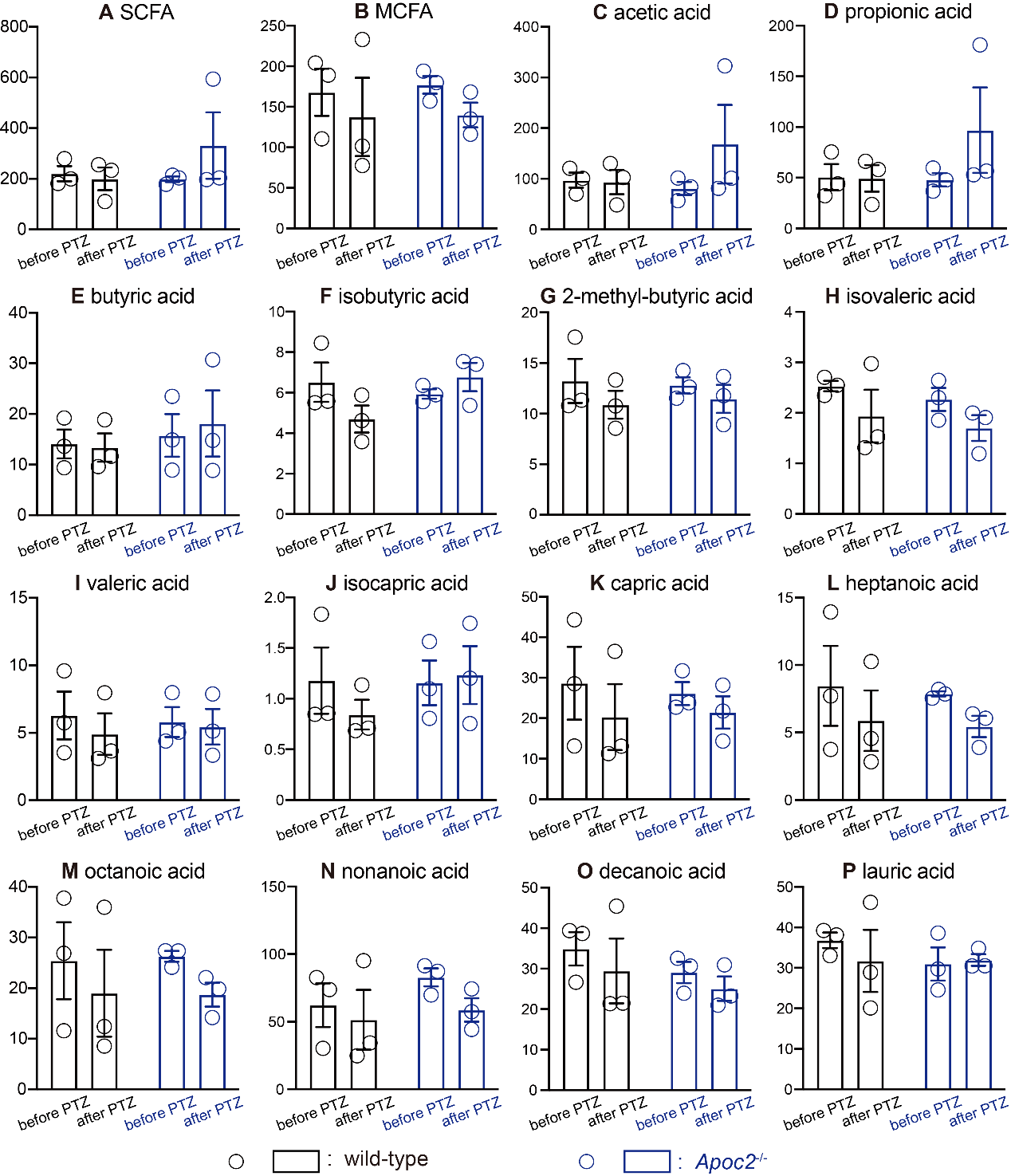

Supplement: SUPPLEMENTARY FIGURE S1 — Statistical graph of short- and medium-chain free fatty acid content in the frontal cortex dialysate of hamsters. Following PTZ-induced seizures, there was no statistically difference in the long-chain free fatty acid levels of both the wild-type hamsters and Apoc2−/− hamsters, including total short- (A), medium-chain free fatty acid (B), acetic acid (C), propionic acid (D), butyric acid (E), isobutyric acid (F), 2-methyl-butyric acid (G), isovaleric acid (H), valeric acid (I), isocapric acid (J), capric acid (K), heptanoic acid (L), octanoic acid (M), nonanoic acid (N), decanoic acid (O) and lauric acid (P). n=3, n represents the number of hamsters recorded in each group. SCFA, short-chain free fatty acid; MCFA, medium-chain free fatty acid. [file Data_Sheet_1.docx]
