# Supplementary material for: Decreased neuronal excitability in hypertriglyceridemia hamsters with acute seizures
Source: Front Neurol. 2024 Dec 19;15:1500737. doi: 10.3389/fneur.2024.1500737 (PMC11730077; doi:10.3389/fneur.2024.1500737)
Supplement: Supplementary file 2 [file Table_1.docx]

**Supplementary Table S1** The number of evoked action potential firings under different current **stimulations.**

| Current(pA) | wild-type (n = 13) | *Apoc2*^-/-^ (n = 15) |
| --- | --- | --- |
| 0 | 0.62 ± 0.33 | 0.13 ± 0.09 |
| +20 | 0.77 ± 0.28 | 0.40 ± 0.24 |
| +40 | 1.92 ± 0.62 | 1.00 ± 0.41 |
| +60 | 3.08 ± 0.70 | 1.73 ± 0.63 |
| +80 | 4.23 ± 0.79 | 2.00 ± 0.63***** |
| +100 | 4.31 ± 0.83 | 2.27 ± 0.72 |
| +120 | 5.46 ± 0.91 | 2.40 ± 0.66***** |
| +140 | 6.00 ± 1.08 | 2.73 ± 0.81***** |
| +160 | 6.23 ± 1.10 | 2.87 ± 0.86***** |
| +180 | 6.54 ± 1.20 | 2.93 ± 1.00***** |
| +200 | 7.00 ± 1.28 | 2.93 ± 0.93***** |
| +220 | 7.31 ± 1.26 | 3.07 ± 0.99***** |
| +240 | 7.08 ± 1.27 | 3.07 ± 1.03***** |
| +260 | 7.00 ± 1.26 | 3.33 ± 1.08***** |
| +280 | 6.92 ± 1.32 | 3.67 ± 1.11 |
| +300 | 6.54 ± 1.43 | 4.07 ± 1.18 |

******P* < 0.05.

**Supplementary Table S2** Statistical table of I_A_ potassium current amplitudes in cortical pyramidal neurons under different voltage stimulations.

| Potential (mV) | wild-type (nA) | *Apoc2*^-/-^ (nA) |
| --- | --- | --- |
| –80 | 0.01 ± 0.00 | 0.06 ± 0.02***** |
| –70 | 0.02 ± 0.00 | 0.09 ± 0.03****** |
| –60 | 0.03 ± 0.01 | 0.12± 0.04***** |
| –50 | 0.045 ± 0.01 | 0.17 ± 0.07***** |
| –40 | 0.07 ± 0.02 | 0.26 ± 0.09***** |
| –30 | 0.10 ± 0.03 | 0.36 ± 0.13***** |
| –20 | 0.17 ± 0.04 | 0.46 ± 0.17***** |
| –10 | 0.23 ± 0.05 | 0.56 ± 0.20 |
| 0 | 0.29 ± 0.06 | 0.68 ± 0.24 |
| +10 | 0.39 ± 0.08 | 0.83 ± 0.22***** |
| +20 | 0.44 ± 0.09 | 1.02 ± 0.21***** |
| +30 | 0.55 ± 0.08 | 1.22 ± 0.17****** |
| +40 | 0.65 ± 0.08 | 1.38 ± 0.15******* |
| +50 | 0.77 ± 0.10 | 1.54 ± 0.16****** |
| +60 | 0.92 ± 0.15 | 1.70 ± 0.18****** |
| +70 | 1.03 ± 0.19 | 1.88 ± 0.19***** |
| +80 | 1.15 ± 0.22 | 2.04 ± 0.22***** |
| +90 | 1.28 ± 0.26 | 2.21 ± 0.25***** |
| +100 | 1.36 ± 0.28 | 2.35 ± 0.28***** |

******P* < 0.05, *******P* < 0.01 and ********P* < 0.001.

**Supplementary Table S3** Statistical table of I_K_ potassium current amplitudes in cortical pyramidal neurons under different voltage stimulations.

| Potential (mV) | wild-type (nA) | *Apoc2*^-/-^ (nA) |
| --- | --- | --- |
| –80 | 0.01 ± 0.00 | 0.02 ± 0.00 |
| –70 | 0.02 ± 0.00 | 0.04 ± 0.01 |
| –60 | 0.03 ± 0.01 | 0.06 ± 0.01 |
| –50 | 0.04 ± 0.01 | 0.10 ± 0.03 |
| –40 | 0.06 ± 0.01 | 0.15 ± 0.04 |
| –30 | 0.10 ± 0.01 | 0.20 ± 0.55 |
| –20 | 0.16 ± 0.04 | 0.32 ± 0.06 |
| –10 | 0.27 ± 0.06 | 0.48 ± 0.08 |
| 0 | 0.36 ± 0.08 | 0.64 ± 0.11 |
| +10 | 0.46 ± 0.10 | 0.83 ± 0.17 |
| +20 | 0.61 ± 0.13 | 1.00 ± 0.23 |
| +30 | 0.77 ± 0.18 | 1.22 ± 0.31 |
| +40 | 0.92 ± 0.21 | 1.40 ± 0.40 |
| +50 | 1.09 ± 0.25 | 1.64 ± 0.48 |
| +60 | 1.25 ± 0.28 | 1.90 ± 0.57 |
| +70 | 1.46 ± 0.34 | 2.13 ± 0.66 |
| +80 | 1.60 ± 0.37 | 2.38 ± 0.74 |
| +90 | 1.79 ± 0.40 | 2.78 ± 0.82 |
| +100 | 1.98 ± 0.44 | 3.11 ± 0.90 |
